# Preservation of Archaeal Surface Layer Structure During Mineralization

**DOI:** 10.1038/srep26152

**Published:** 2016-05-25

**Authors:** Adrienne Kish, Jennyfer Miot, Carine Lombard, Jean-Michel Guigner, Sylvain Bernard, Séverine Zirah, François Guyot

**Affiliations:** 1 Molécules de Communication et Adaptation des Microorganismes, Sorbonne Universités, Muséum National d’Histoire Naturelle, CNRS UMR 7245; CP54, 57 rue Cuvier 75005 Paris, France; 2 Institut de Minéralogie, de Physique des Matériaux et de Cosmochimie, Sorbonne Universités, Muséum National d’Histoire Naturelle, UMR CNRS 7590, Université Pierre et Marie Curie, IRD UMR 206, 61 rue Buffon/4 place Jussieu, F-75005 Paris, France

## Abstract

Proteinaceous surface layers (S-layers) are highly ordered, crystalline structures commonly found in prokaryotic cell envelopes that augment their structural stability and modify interactions with metals in the environment. While mineral formation associated with S-layers has previously been noted, the mechanisms were unconstrained. Using *Sulfolobus acidocaldarius* a hyperthermophilic archaeon native to metal-enriched environments and possessing a cell envelope composed only of a S-layer and a lipid cell membrane, we describe a passive process of iron phosphate nucleation and growth within the S-layer of cells and cell-free S-layer “ghosts” during incubation in a Fe-rich medium, independently of metabolic activity. This process followed five steps: (1) initial formation of mineral patches associated with S-layer; (2) patch expansion; (3) patch connection; (4) formation of a continuous mineral encrusted layer at the cell surface; (5) early stages of S-layer fossilization via growth of the extracellular mineralized layer and the mineralization of cytosolic face of the cell membrane. At more advanced stages of encrustation, encrusted outer membrane vesicles are formed, likely in an attempt to remove damaged S-layer proteins. The S-layer structure remains strikingly well preserved even upon the final step of encrustation, offering potential biosignatures to be looked for in the fossil record.

Structure and chemical composition of cell envelopes are defining characteristics of the main branches of prokaryotic microorganisms. They determine for example which molecules are permitted to penetrate the cell interior, the mechanical strength available to counteract stress conditions, and the nature of interactions with metals in the environment. One cell envelope component common to representatives of most major phylogenetic branches of prokaryotes is a proteinaceous surface layer, or S-layer. The simplicity of S-layers as cell walls, particularly in certain archaeal species for which the S-layer is the sole component of their cell envelope outside the lipid membrane, has led to the suggestion that S-layers may have been an early form of cell wall in the evolution of prokaryotes^1^. The S-layer structure is usually composed of a single main protein type (40–200 kDa) arranged into a highly ordered structure. These rigid two-dimensional crystalline arrays of identical proteins are capable of self-assembly, both on cell surfaces, and in solution *in vitro* as cell-free S-layer “ghosts”^2^. The β-sheet structure of S-layer protein monomers is highly conducive to self-assembly of the repeating, ordered structure^3^.

S-layer proteins are among the most abundant biopolymers on Earth, representing 15% of all cellular proteins in bacterial cells^4^. S-layers are anchored either directly into the lipid membrane as is the case in the Archaea, or to peptidoglycan via secondary cell wall polymers as in Gram-positive bacteria, or associated with the lipopolysaccharide outer membrane of Gram-negative bacteria^2^. The fact that both mesophilic and extremophilic bacteria and archaea possess S-layers indicates that they represent a common evolutionary adaptation against a range of stresses. S-layers augment the structural stability of prokaryotic cells, thereby protecting cells against predation^5^ and stabilizing lipid membranes under environmental stresses such as high temperature, mechanical stress^6^, or osmotic and hydrostatic pressures^7^,^8^. They also serve as a molecular sieve on top of the semi-permeable lipid membrane, as well as aid in cell-to-cell and cell-to-surface adhesion^9^. The survival advantages provided by S-layers have likely contributed their maintenance by diverse prokaryotes despite the high metabolic costs of producing such an organic “exoskeleton”.

S-layers are capable of adsorbing metals and radionuclides, including Pd(II), Cu(II), Pt(II), Au(III)^10^ and U(VI) in the form of uranyl phosphate^11^,^12^. Metals interactions with S-layers can result in altered physical properties of both the metals and the S-layers themselves, including the deposition of Au-nanoparticles on S-layers resulting in increased structural stability of the S-layers and augmented paramagnetism of Au nanoparticles^13^,^14^. S-layers from cyanobacteria have been described as the nucleation site of carbonate and sulfate minerals including fine-grained gypsum (CaSO_4_.2H_2_O), calcite (CaCO_3_)^15^, strontianite (SrCO_3_) and celestite (SrSO_4_)^16^. Over twenty years have passed since these pioneering studies, and yet the mechanisms of S-layer mineralization remain poorly constrained.

Here, we report observations of the hyperthermophilic archaeon *Sulfolobus acidocaldarius*. This strain was originally isolated from metal-rich, acidic solfatares and hot springs. It is an aerobic heterochemoorganotroph with small, lobed cocci cells 1–2 μm in diameter, growing at temperatures between 55–85 °C under acidic conditions (pH 1–6) in the presence of sulfur and a variety of metals including iron^17^. The cell envelope in the *Sulfolobales* only consists of a S-layer anchored to the cell membrane. The S-layer of *S. acidocaldarius* is composed of two proteins; SlaA, which forms the S-layer surface and SlaB, which forms a pedestal extending out from the lipid membrane to anchor the SlaA protein lattice^18^, creating a quasi-periplasmic space (or quasi-periplasm)^19^. The basic construction of the S-layer in *S. acidocaldarius* consists in a repeating lattice of SlaA protein monomers supported by the pedestal-like SlaB protein anchoring it to the cell membrane^18^. These S-layers may provide nucleation sites for mineral precipitation.

Here, iron being prevalent in the natural environment of *Sulfolobale* species^20^, we focused on nucleation and growth of Fe-bearing minerals in contact with the S-layer of *S. acidocaldarius*, iron being prevalent in the natural environment of *Sulfolobale* species^20^. We performed conventional and cryo-TEM analyses on experimentally biomineralized whole cells and cell-free S-layer “ghosts”. Incubation of these samples in a Fe-rich medium induced the formation of crystalline iron oxyhydroxide and amorphous iron phosphate minerals in close association with S-layers.

## Results

### Nature of minerals associated with S-layers

**Figure 1:**
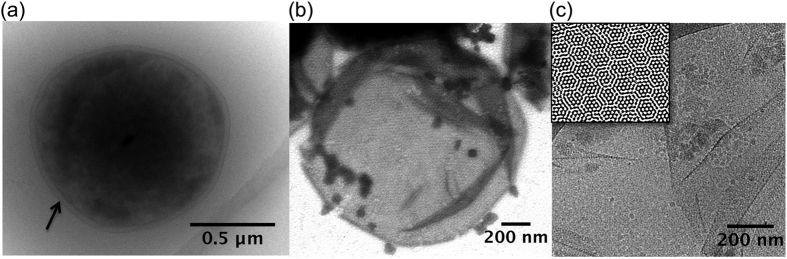
S-layer structure of *Sulfolobus acidocaldarius* from cryoTEM observations of whole cells [Panel (**a**); arrow points to S-layer] and reconstituted S-layer extracts [S-layer ghosts, conventional TEM observations after negative staining using 1% sodium phosphotungstate in Panel (**b**) and cryoTEM observations in Panel (**c**)]. Panel (**c**) insert shows inverse fast Fourier transform (IFFT) image analyses confirming a p3 hexagonal S-layer symmetry.

**Figure 2:**
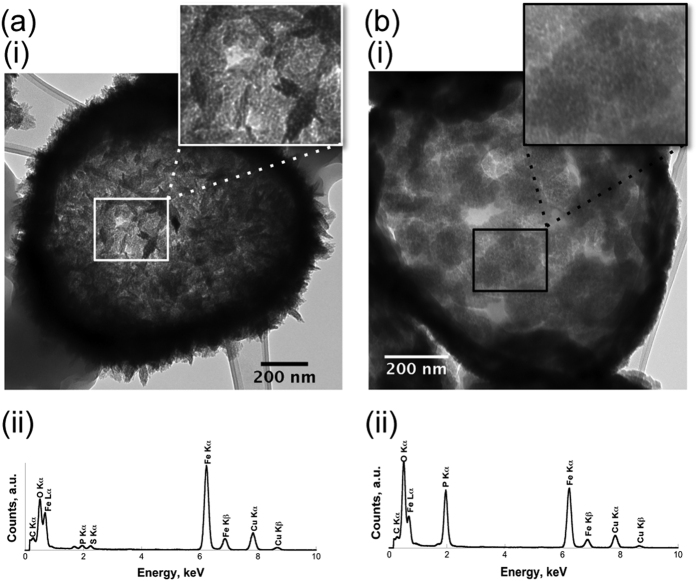
Comparison of *S. acidocaldarius* S-layer biomineralization patterns obtained after 16 h incubation in the presence of only FeSO_4_ [Panel (**a**)] or a mix of FeSO_4_ and NaH_2_PO_4_ [Panel (**b**)]. TEM observations (i), and elemental analysis by EDXS (ii). Minerals formed on the cell surfaces corresponded to nanocrystalline ferric iron (oxy)hydroxides (**a**) or amorphous Fe phosphates (**b**). Only in the case of Fe phosphate formation was the hexagonal p3 symmetry of the *S. acidocaldarius* S-layer made visible in mineral patches [Panel **b**(i)]. Minor P and S peaks present in the EDX spectra in Panel (**a**) likely correspond to components of the *S. acidocaldarius* cell envelope, rather than minerals.

**Figure 3:**
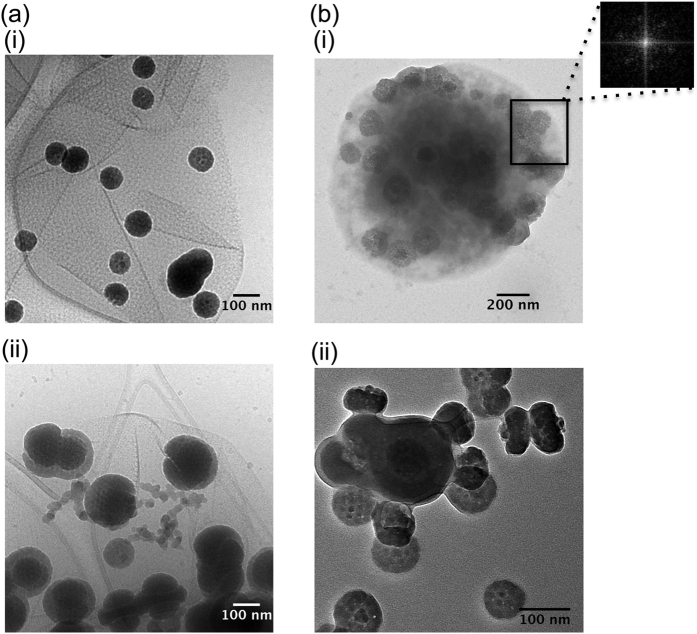
Iron phosphate formation at the contact of S-layers from *S. acidocaldarius* showing identical mineral formation in both cell-free S-layer ghosts [Panel (**a**); cryoTEM] and whole cells [Panel (**b**); conventional TEM (i) and cryoTEM (ii)]. Images correspond to Steps #2–3 of mineral formation as presented in Fig. 5. Inset in Panel **b**(i) is the fast Fourier transform (FFT) pattern of the boxed mineral patches, exhibiting the p3 hexagonal symmetry.

Cell envelopes in *Sulfolobus* are extremely simple, being composed only of a cell membrane (tetraether lipids) and a surrounding proteinaceous S-layer that are easily identified in the cryo-TEM images of whole cells (Fig. 1a). Inverse Fast Fourier transforms of images of S-layer ghosts (Fig. 1b,c and insert) confirmed that these cell-free extracts of S-layer proteins retained not only the size and shape of the cell, but also the hexagonal (p3) S-layer symmetry previously described for this species^21^. In a phosphate-free medium enriched in FeSO_4_ in the presence of whole cells, nanocrystalline needles of a few tens of nanometers were precipitated in association with *S. acidocaldarius* cells (Fig. 2a). In contrast, in phosphate- and sulfate-rich media (FeSO_4_ + NaH_2_PO_4_), P- and Fe-rich particles precipitated by whole *S. acidocaldarius* cells as globules (Fig. 2b(i)). EDXS analyses (Fig. 2a(ii)) and SAED patterns (data not shown) were consistent with crystalline ferric iron (oxy)hydroxides, such as goethite (α-FeOOH) (Fig. 2a), and amorphous Fe phosphates (Fig. 2b). While FeOOH phases completely hid the S-layer structure, Fe phosphate formation replicated the S-layer structure, thereby enhancing the contrast of the hexagonal S-layer symmetry (Figs 2b(i) and 3). The encrustation patterns of whole cells and cell-free S-layer ghosts containing SlaA and SlaB proteins (see Supplementary Information and Supplementary Fig. S1) appeared identical (Fig. 3; see also Supplementary Fig. S2), thereby confirming that the S-layers are a preferred site of mineral formation, independent of metabolic processes associated with whole, living cells.

### Steps of mineral formation and cell wall encrustation

**Figure 4:**
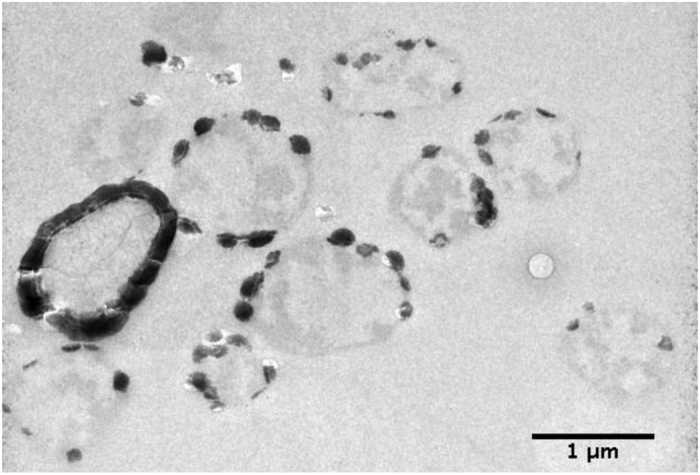
Coexistence of cells at different stages of biomineralization/mineral encrustation after 24 h in Fe-rich medium.

**Figure 5:**
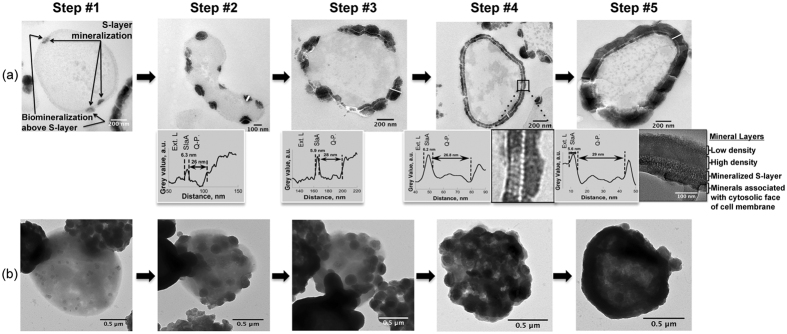
The successive steps of S-layers encrustation by Fe phosphate in *S. acidocaldarius* imaged by TEM of ultrathin sections [Panel (**a**)] and whole cells [Panel (**b**)]. Step #1 – Initial formation of mineral patches associated with S-layer; Step #2 - Expansion of mineral patches. Mineral buildup forms as a dome-like structure (extracellular layer, Ext. L.) over initial patches of S-layer/mineral assemblies (S-L.); Step #3 - Connection of mineral patches (Ext. L. and S-L.); Step #4 – Formation of a continuous mineral encrusted layer at the surface of the entire cell (inset below is a larger view of the boxed region showing electron-bright and electron-dark spots in the mineralized S-layer); Step #5 - Early stages of S-layer fossilization via growth of the extracellular mineralized layer (Ext. L.) and the mineralization of cytosolic face of the cell membrane below the S-layer (insert below shows details of the cell wall during early fossilization revealing a highly electron-dense layer directly above the S-layer with a less dense layer on top). All images were of unstained samples, with the exception of micrographs shown in Panel (**a**) for Steps #1&2, which correspond to samples stained with saturated aqueous uranyl acetate. Histograms next to micrographs in Panel (**a**) give the intensity of electron density along the profiles (shown as white segments) across the mineral-encrusted cell wall. Ext. L.: extracellular mineral layer; Q-P.: quasi-periplasmic space; SlaA.: SlaA S-layer protein; S-L.: S-layer/mineral assemblies.

Formation of Fe phosphate on the cell surface occurred after only 3 h of incubation in the Fe-rich medium. Importantly, whatever the incubation time, cells exhibiting different levels of encrustation, from slightly mineralized (described as Step #1 in the following) to heavily mineralized (described as Step #5 in the following) coexisted (Fig. 4). Mineral formation proceeded through five steps, as shown in Fig. 5.

Step #1 was the formation of patches of a few tens of nanometers in length (85+/−2 nm, n = 14, where ‘n’ is the number of independent features measured) onto discrete sites within the S-layer and quasi-perisplasmic space, which resulted in a slight deformation of the cell wall. Mineral formation then proceeded above the S-layer through the formation of a dome-like structure of varying thickness (Step #2, extracellular mineral-encrusted layer), which intensified the concave deformation of the S-layer. During Step #3, these mineral patches multiplied, expanded, and eventually connected, leading to a more or less continuously mineral encrusted S-layer. In Step #4, a continuous extracellular mineral layer was formed surrounding the completely mineral encrusted S-layer. Finally, in Step #5 the thickness and density of the extracellular mineral layer increased, while mineral phases precipitated at the contact of the cytosolic face of the cell envelope. This final stage of mineral formation affected cell integrity through increased cell lysis.

The mineral encrusted cell wall exhibited a very specific texture. During the first steps of mineral formation, the p3 hexagonal symmetry of the S-layer was preserved [Figs 2b(i) and 3a(i),b(ii)]. Of note, TEM observations of cross-sections of *S. acidocaldarius* cells at more advanced steps of mineral formation revealed the coexistence of electron-dense and electron-light spots found to be on average 6 nm in diameter (+/−1 nm, n = 14) (see Fig. 5, Step #4 boxed insert). We interpret electron-light spots as mineral-free SlaA protein monomers marking the upper boundary of the mineral encrusted quasi-periplasm (Q.P.). This Q.P. is created by the height of the SlaB protein separating the SlaA array from the lipid membrane, and did not vary between non-mineralized and mineralized regions of the cell envelope [27 nm-thick (+/−4 nm, n = 10) for non-mineralized regions and 27 nm-thick (+/−3 nm, n = 15) for mineralized regions]. In addition, a more electron-dense extracellular mineral layer covers the cell wall (see histograms in Fig. 5, Steps #2–5). At advanced stages of mineral formation, the electron-light zone hypothesized to be SlaA resolves into distinct circular units, evenly distributed with an average distance of 11 nm (+/−1 nm, n = 25) between the centers of each unit (see Fig. 5, Step #4). The present measurements are in good agreement with those of SlaA previously reported for the related species *Sulfolobus shibatae*, the only other member of the Sulfolobale for which such measurements are available^22^,^23^. The thickness of the quasi-periplasmic space of *S. acidocaldarius*, however, was 75% larger than that reported for *S. shibatae*^23^.

### Vesicle production upon encrustation

**Figure 6:**
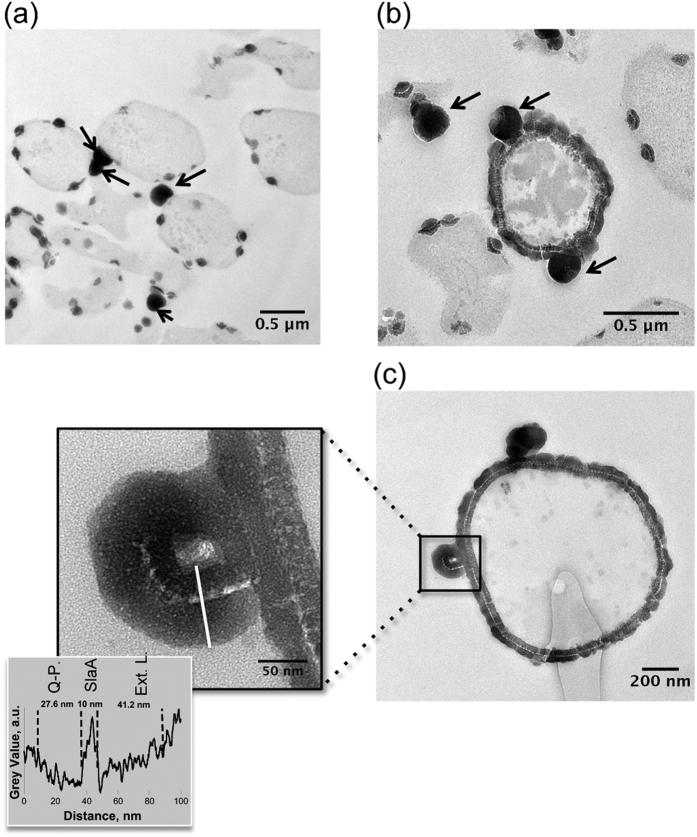
Vesicle formation and release as a stress response to S-layer mineralization in *S. acidocaldarius* visualized by TEM analyses of ultra-thin sections [Panels (**a**,**b**); arrows point to vesicles]. Panel (**c**) insert shows magnification of a vesicle with a completely mineral-encrusted S-layer with a profile of electron density (grey value) across the vesicle. Ext. L.: extracellular mineral layer; Q-P.: quasi-periplasmic space; SlaA.: SlaA S-layer protein.

Outer membrane vesicles of 175 +/−5 nm in diameter (n = 24) formed and were released concomitantly to S-layer encrustation (Fig. 6). Interestingly, these vesicles were fully encrusted by minerals, even when cells were only partially encrusted. They exhibited the same texture as the encrusted cell wall of whole cells, which we interpreted as follows: a 28 nm-thick layer corresponding to the encrusted quasi-periplasm, a 10 nm-thick electron light layer corresponding to the SlaA layer and a thick (41 nm-thick) external layer corresponding to extracellular minerals (Fig. 6c).

## Discussion

Prokaryotes (Bacteria and Archaea) represent the most ancient lineages of life on Earth. Their evolution is intimately linked to that of their surrounding geochemical environment, e.g. as a source of essential metal ions for cellular processes. Metals may be transported across membranes, transformed through redox reactions, adsorbed, and/or precipitated as minerals either through passive biologically induced biomineralization or active biologically controlled biomineralization mechanisms^24^,^25^. Nucleation of mineral phases can occur at both intracellular and extracellular locations, sometimes as one of many mechanisms used to reduce the toxic effects of high metal concentrations. Whereas bacteria encrustation by iron minerals has most often been explored in the context of microbial Fe-based metabolisms^26^,^27^,^28^,^29^,^30^, encrustation of microorganisms, particularly by iron minerals, has been observed in hydrothermal environments as well^31^,^32^.

Here we show that iron-bearing minerals precipitate on the surface of *S. acidocaldarius* cells and cell-free S-layer ghosts during incubation in a Fe-rich medium, demonstrating that this mineral formation is a “passive” process not reliant on any cellular metabolic activities. The initial formation of mineral patches (Step #1, Fig. 5) appears to occur within the S-layer pore spaces and the quasi-periplasmic space. A recent analysis of the interactions of S-layers and metals *in vacuo* using X-ray absorption and photoemission spectroscopies revealed that iron reacts directly with carboxyl and hydroxyl groups of the amino acids composing the S-layer protein of *Lysinibacillus sphaericus* NCTC 9602, leading to the formation of metal-protein complexes through metal oxidation reactions^33^. Metal cations may also interact directly with negatively charged molecules in the cell envelope^10^, including the carboxyl and hydroxyl functional groups of the amino acids of both SlaA and SlaB proteins. After incubation within the Fe-rich medium, *S. acidocaldarius* cells and S-layer ghosts exhibit very similar biomineralization patterns (Fig. 3). This shows that this process is purely “abiotic” (non-metabolic). Such a nucleation process appears similar to the one described for the fossilization of cyanobacteria by fine-grained gypsum (CaSO_4_.2H_2_O)^15^,^16^, though under different chemical conditions.

After nucleation within the S-layer pores spaces, encrustation expands to larger patches of the S-layer with the concurrent formation of dome-like structures over the S-layers (Step #2, Fig. 4). The connection of discrete dome-like patches (Step #3, Fig. 5) eventually leads to the formation of a continuous mineral layer over the entire cell surface (Step #4, Fig. 5). Of particular importance, the S-layer physical structure seems to be preserved during mineral formation and growth at its contact.

In addition to this structural preservation, the present study reports the formation of extracellular membrane vesicles during incubation within the Fe-rich medium. While the production of such vesicles is known to be a common feature of hyperthermophilic archaea, including deep-sea vent *Thermococcales* spp.^34^,^35^ and *Aciduliprofundum boonei*^36^, shallow and deep-sea hydrothermal vent *Pyrococcus* spp.^35^, and solfatara and hot spring-inhabiting *Sulfolobus* spp.^37^, the present study constitutes the first report of vesicle production as a response to S-layer encrustation by minerals.

Like the cell walls from which they originate, extracellular membrane vesicles in the Sulfolobales possess S-layers and are thought to be formed by an active budding mechanism based on Endosomal Sorting Complex Required for Transport III (ESCRT-III) proteins^38^. The fact that the vesicles observed here were found to be encrusted by minerals prior to their release from the cell of origin suggests that the S-layer flexibility is maintained even while encrusted, thereby permitting the curvature of the S-layer required for vesicle budding. The structure of the S-layer indeed allows it to bend. Three-dimensional image reconstruction of the structure of the SlaA S-layer protein from *S. acidocaldarius* by Taylor *et al.* revealed three globular domains connected by hinge-like narrow bridges allowing curvature of the S-layer array, while the supporting pedestal-like SlaB proteins anchoring the S-layer to the cell membrane likely prevent overcrowding of the interior surface of the SlaA protein subunits during cell wall bending^39^.

Prokaryotes possessing S-layers are widespread in metal-rich (e.g. ferruginous) environments. As S-layers become passively encrusted with Fe minerals, one may wonder why and how such features persisted through evolution. On the one hand, either induced or passive encrustation by iron minerals has been suggested to provide some advantages to microorganisms: metabolic (Fe minerals may serve as sinks and sources of Fe for chemolithoautotrophy^40^,^41^) or physiological (Fe minerals may serve as a protective shield against UV radiation^42^). On the other hand, prokaryotes may have developed strategies to deal with such mineralization. Phoenix *et al.*^43^ have shown that the viability of encrusted cyanobacterial cells was made possible by extracellular sheath encrustation. *Synechococcus* GL24 inhabiting alkaline lake waters are hypothesized to shed their extracellular sheath once being heavily encrusted^15^,^44^. This appears very similar to the formation of vesicles by *S. acidocaldarius* cells described in the present study. Such production of extracellular membrane vesicles can be seen as a response to environmental stresses, including exposure to potentially toxic metals such as iron. Makarova *et al.*^33^ demonstrated that metal oxidation reactions, particularly with Fe, ultimately denature S-layer proteins. Membrane vesicle release may therefore be seen as an attempt by *Sulfolobus* cells to eliminate damaged S-layer proteins in order to enable replacement with new proteins (not associated with minerals), in a manner similar to that seen in organisms lacking S-layers for elimination of denatured or mineral-associated cell wall components^45^,^46^. Based on our present results, this issue of cell viability and response to stress upon passive Fe biomineralization would deserve a dedicated study.

## Conclusions

**Figure 7:**
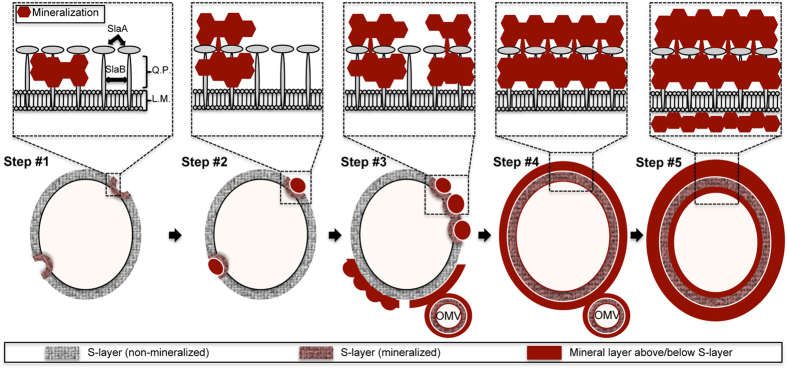
Schematic of the steps in S-layer encrustation by Fe-phosphate with inset images showing a detailed schematic representation of the cell envelope at each stage of mineral formation: (Step #1) initial formation of mineral patches associated with S-layer in the quasi-periplasm (Q.P.) formed between the lipid membrane (L.M.) and the SlaA S-layer array (SlaA) supported by SlaB pedestals (SlaB), (Step #2) expansion of mineral patches and mineral buildup forming dome-like structures over initial patches of S-layer mineralization, (Step #3) connection of mineral patches, (Step #4) formation of a continuous mineral encrusted layer encrusting the entire cell surface, leaving a non-mineralized layer corresponding to the SlaA S-layer array, and (Step #5) early stages of S-layer fossilization via growth of the extracellular mineralized layer and the mineralization of cytosolic face of the cell membrane below the S-layer. At more advanced stages of encrustation (Steps #3 and 4), encrusted outer membrane vesicles (OMV) are formed.

The present study demonstrates that the structure of S-layers in *S. acidocaldarius* may be preserved despite the precipitation of iron phosphates. This passive process of Fe phosphate nucleation and growth within the S-layer pore spaces can be described in five steps leading to the complete encrustation of the cell wall and the cytosolic face of the cell membrane (Fig. 7). Mineral formation in association with S-layers could be the driving force for the production of extracellular membrane vesicles in an attempt to remove damaged S-layer portions. Of note, even upon the final step of encrustation (Step #5, Fig. 5), the S-layer structure is strikingly well preserved [shown in inset below Step #5 in Fig. 5]. Importantly, such S-layer structure preservation was only observed in the case of Fe phosphate formation and not in the case of Fe oxyhydroxide formation (Fig. 2). Ultrastructural details and molecular signatures may persist upon diagenesis, as shown by recent studies of natural samples^47^,^48^,^49^,^50^,^51^,^52^ as well as experimental studies 29,53,54,55,56. Microbial remains have been shown to be sometimes very well preserved upon early stages of fossilization^57^,^58^, in particular in Fe-rich media^55^,^59^. The fine features of S-layers described in the present study may thus constitute pertinent biosignatures to be looked for in the geological record, as long as they would be preserved upon diagenetic or metamorphic conditions. This undoubtedly deserves further investigation.

## Methods

### Microbial Culturing Conditions and Mineralization

*S. acidocaldarius* is an obligate aerobe naturally found in mineral-rich acidic hot springs and solfatara soils. While environmental isolates of *S. acidocaldarius* may grow either heterotrophically or autotrophically through the oxidation of elemental sulfur to sulfuric acid under high temperature and low pH conditions^17^, the DSM 639 type strain has apparently lost the capacity for autotrophic growth and can only grow in the presence of an organic carbon source such as yeast extract^20^. In the present study, *Sulfolobus acidocaldarius* DSM 639 cells were cultured at 80 °C aerobically in Brock’s medium^17^ pH 3.5 supplemented with 0.1% yeast extract and 0.2% D-saccharose under 190 rpm agitation, resulting in an average generation time of 6 h. Fe concentration in this growth medium was very low (73 μM FeSO_4_). Cells were grown to mid-exponential growth phase (OD_600nm_ = 0.6) and harvested by centrifugation (8000 × g, 10 min). Cells were then washed twice in MilliQ water and resuspended in the appropriate Fe-rich medium (10 mM FeSO_4_ pH 4.5, or 10 mM FeSO_4_ and 10 mM NaH_2_PO_4_ pH 4.5) at a density of approximately 3.5 × 10^10^ cells/mL. All cultures as well as abiotic controls (not inoculated with *S. acidocaldarius* DSM 639 culture) were incubated under aerobic conditions at 60 °C, 150 rpm for 3–24 h. Cells were harvested by centrifugation (8000 × g, 10 min) and washed twice in MilliQ water.

### S-layer extraction and mineral precipitation procedure

*S. acidocaldarius* S-layers were extracted according to the protocol reported by Reitz *et al.*^60^. Briefly, *S. acidocaldarius* cells cultured in Brock’s medium were harvested by centrifugation (8000 × g, 10 min), resuspended in an extraction buffer [HEPES buffer (pH 7.4) containing 2 mM EDTA, 0.15% SDS] containing 50 μg/mL DNase I, and incubated at room temperature with gentle agitation for 1 h. The SDS concentration was then increased to 2% prior to overnight incubation at room temperature with gentle agitation, followed by centrifugation (40 000 × g, 30 min). The resulting pellet was washed a total of three times in extraction buffer by incubation at 60 °C with gentle agitation for 1 h. The insoluble fraction was then collected by centrifugation (40 000 × g, 30 min). The resulting S-layer ghosts were washed eight times in MilliQ water to remove all traces of SDS. SDS-PAGE and mass spectrometry analyses verified that the S-layer ghosts contained both the SlaA S-layer protein and the SlaB pedestal protein supporting the SlaA S-layer array (see Supplementary Information, Supplementary Fig. S1).

Whole cells and S-layer ghosts were transferred into the Fe-rich medium under hydrothermal conditions (60 °C) and acidic pH with gentle agitation to provide aeration simulating the natural environment: suspensions were placed in the appropriate Fe-rich medium (10 mM FeSO_4_ pH 4.5, or 10 mM FeSO_4_ and 10 mM NaH_2_PO_4_ pH 4.5) and incubated under aerobic conditions at 60 °C, 150 rpm for different durations up to 24 h. Minerals associated with whole cells and S-layer ghosts were harvested by centrifugation (8000 × g, 30 min) and washed twice in MilliQ water.

### Transmission Electron Microscopy (TEM)

For conventional TEM analyses, samples were either directly deposited onto 100-mesh formvar/carbon coated copper grids after washing in MilliQ water, or fixed in 0.1 N sodium cacodylate buffer (pH 7.4) containing 2.5% gluteraldehyde at 4 °C. Fixed samples were used to prepare ultrathin sections by ultramicrotomy. Cells were post-fixed for 1 h through the addition of 1% OsO_4_, followed by three washes in 0.1 N sodium cacodylate buffer (pH 7.4), dehydration in increasing concentrations of ethanol, and progressive embedding in Spurr resin. Ultrathin sections (70-nm thick) were cut with a Reichert-Jung Ultracut E ultramicrotome and deposited onto 100-mesh formvar/carbon coated copper grids. Some sample grids were stained prior observation with saturated aqueous uranyl acetate. S-layer ghosts were deposited directly onto grids and negatively stained using 1% sodium phosphotungstate.

CryoTEM analyses were also employed to observe the S-layer structure under aqueous (“near-native”) conditions, with a reduction in alteration/modification of bacterial cell structures that can occur during conventional TEM preparations, especially upon dehydration^61^. The concomitant low contrast of the cryo-TEM images can occur due to high sample thickness, but provides very detailed ultrastructural information^62^,^63^. Enhanced contrast is however obtained for biomineralized samples^60^. For these analyses, whole cells were concentrated by gentle centrifugation (6000× g, 10 min), resuspended in HEPES buffer (pH 7.4). A drop of either whole cells or S-layer ghosts was deposited on a “quantifoil”^®^ (Quantifoil Micro Tools GmbH, Germany) carbon membrane. The excess of liquid on the membrane was blotted out with a filter paper and the membrane was quench-frozen before evaporation in liquid ethane, to form a thin vitreous ice film in which either whole cells or S-layer ghosts were captured. Conventional and cryo-TEM experiments were performed using a JEOL2100 microscope equipped with a LaB_6_ electron source and operating at 200 kV. Cryo-TEM observations were performed at −180 °C under low-dose conditions (less than 10 electrons Å^−2^ s^−1^) at nominal magnifications of 20 000 and 40 000. Images were recorded on a 2 k by 2 k pixels CCD camera (Gatan Ultrascan 1000). Elemental analyses were carried out using Energy Dispersive X-ray Spectroscopy (EDXS). Minerals were identified based on their chemical composition and selected area electron diffraction (SAED) patterns. S-layer symmetry was determined through Inverse Fast Fourier Transform (IFFT) image analyses of selected areas in TEM images.

## Additional Information

**How to cite this article**: Kish, A. *et al.* Preservation of Archaeal Surface Layer Structure During Mineralization. *Sci. Rep.* **6**, 26152; doi: 10.1038/srep26152 (2016).

## Supplementary Material

Supplementary Information
