# Supplementary material for: Preservation of Archaeal Surface Layer Structure During Mineralization
Source: Sci Rep. 2016 May 25;6:26152. doi: 10.1038/srep26152 (PMC4879539; doi:10.1038/srep26152)
Supplement: Supplementary Information [file srep26152-s1.pdf]

Preservation of Archaeal Surface Layer Structure During Mineralization

Adrienne Kish[1*], Jennyfer Miot[2], Carine Lombard[1], Jean-Michel Guigner[2], Sylvain Bernard[2], Séverine Zirah[1], François Guyot[2]

[1] Molécules de Communication et Adaptation des Microorganismes, Sorbonne Universités, Muséum National d'Histoire Naturelle, CNRS UMR 7245; CP54, 57 rue Cuvier 75005 Paris, France

[2] Institut de Minéralogie, de Physique des Matériaux et de Cosmochimie, Sorbonne Universités, Muséum National d'Histoire Naturelle, UMR CNRS 7590, Université Pierre et Marie Curie, IRD UMR 206, 61 rue Buffon/4 place Jussieu, F-75005 Paris, France

* Correspondence and requests for materials should be addressed to A.K. (adrienne.kish@mnhn.fr)

SUPPLEMENTARY INFORMATION

To verify the protein complement of the S-layer ghosts, we treated the prepared S-layer ghost extract for 20 min at 45 ^{o}C in sodium carbonate buffer (20 mM) to ensure complete solubilization of proteins prior to their electrophoretic separation under denaturing conditions using Sodium Dodecyl Sulphate Polyacrylamide Gel Electrophoresis (SDS-PAGE; 10 % separating gel)[1]. After staining with Coomassie Brilliant Blue G-250, two bands were observed, as seen in Supplementary Fig. S1, corresponding to the expected molecular masses of SlaA (approximately 150 kDa) and SlaB (approximately 60 kDa). The 150 kDa band was excised, subject to reduction with dithiothreitol (DTT, 10 mM), alkylation with iodoacetamide (55 mM) and digestion with trypsin (20 ng/µL) and then analyzed by mass spectrometry as previously described[2]. The LC-MS analysis was conducted on an Ultimate 3000 Micro-HPLC system (Thermo Scientific) connected to an electrospray ionization (ESI)-hybrid quadrupole time-offlight (QqTOF) QSTAR Pulsar mass spectrometer (AB Sciex) equipped with an ion spray source. The MS instrument was operated in positive mode using information–dependent acquisition (IDA). This mode completes a series of 1-s survey scan (*m/z* detection range, 250 to 1300) followed by two 2-s tandem MS (MS-MS) experiments on the two most intense peaks from the survey scan, as long as they have not been fragmented in the last 2 min (*m/z* detection range, 50 to 1500). The data generated were analyzed with Mascot (http://www.matrixscience.com/), and proteins identified by comparison of the data to the NCBInr database. Five peptides corresponding to the SlaA S-layer protein (GenBank accession number gi|499598404) were detected (Fig. S1).

The minerals formed on the surfaces of whole cells of *S. acidocaldarius* and cell-free S-layer "ghosts" appeared to be the same (see Fig. 3). This finding was confirmed by the corresponding EDX spectra, as shown in Figure S2.

Figure S1. Identification of S-layer ghost proteins. (a) SDS-PAGE of S-layer ghost extracts; with two bands corresponding to the expected molecular masses of SlaA (150 kDa) and SlaB (60 kDa). (b) LC/MS results showing the set of peptides identified (Mr exp: experimental monoisotopic mass, Mr calc: monoisotopic mass calculated from the matched peptide sequence, Sequence: sequence of the peptides and neighboring amino acids, separated with dots).

(a)

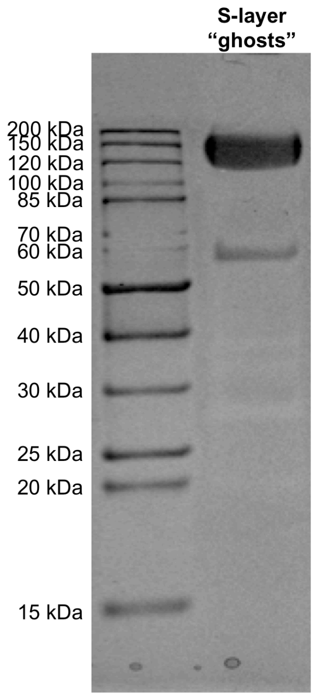

(b)

**Mr(expt)**	**Mr(calc)**	**Sequence**
1213.7737	1213.7646	K.LVALSTLSLVAK.G
1574.7518	1574.7439	K.QPFMTYSDLQFAK.T
1590.7651	1590.7388	K.QPFMTYSDLQFAK.T + Oxidation (M)
1786.0397	1786.0141	R. LAPFQVPAQVPLPNAPK.L
2567.3699	2567.3119	K.LVVEYPSSTNFLPQGVTVTTSSNK.Y

Figure S2. Comparison of EDX spectra of precipitates formed on *S. acidocaldarius* DSM 639 whole cells [Panel (a)] and S-layer “ghosts” [Panel (b)] after 16 h incubation in the presence of a mix of $FeSO_4$ and NaH_2PO_4.

(a) (b)

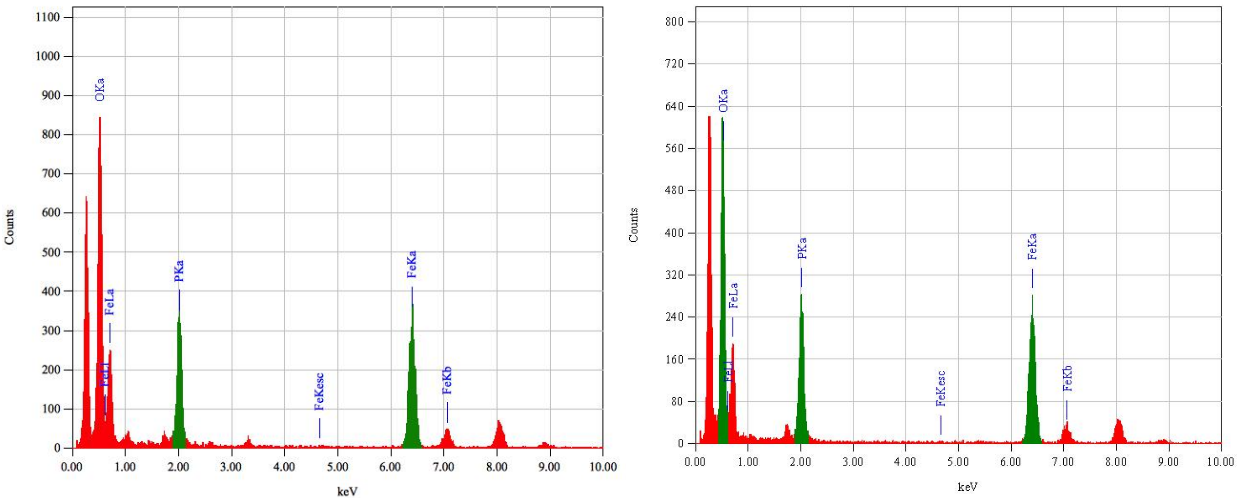

REFERENCES

1. Reitz, T. *et al.* Spectroscopic study on uranyl carboxylate complexes formed at the surface layer of Sulfolobus acidocaldarius. *Dalton Transactions* **44,** 2684-2692 (2015).
2. François, F. *et al.* Isolation and characterization of environmental bacteria capable of extracellular biosorption of mercury. *Appl Environ Microbiol* **78,** 1097–1106 (2012).
